# Proteomic Analysis of the Characteristic Flavor Components in *Bacillus subtilis* BSNK-5-Fermented Soymilk

**DOI:** 10.3390/foods13152399

**Published:** 2024-07-29

**Authors:** Miao Hu, Jiao Wang, Yaxin Gao, Bei Fan, Fengzhong Wang, Shuying Li

**Affiliations:** 1 Institute of Food Science and Technology, Chinese Academy of Agricultural Sciences, No. 2 Yuan Ming Yuan West Road, Beijing 100193, China; humiao7890@163.com (M.H.); wangjiao202309@163.com (J.W.); gaoyx1997@163.com (Y.G.); fanbei517@163.com (B.F.); 2 Key Laboratory of Agro-Products Quality and Safety Control in Storage and Transport Process, Ministry of Agriculture and Rural Affairs, Chinese Academy of Agricultural Sciences, Beijing 100193, China; 3 Key Laboratory of Agro-Products Processing, Ministry of Agriculture and Rural Affairs, Chinese Academy of Agricultural Sciences, Beijing 100193, China

**Keywords:** *Bacillus subtilis*, fermented soymilk, characteristic flavor, proteomics, TMT technology

## Abstract

Fermentation with *Bacillus subtilis* significantly enhances the physiological activity and bioavailability of soymilk, but the resulting characteristic flavor seriously affects its industrial promotion. The objective of this study was to identify key proteins associated with characteristic flavors in *B. subtilis* BSNK-5-fermented soymilk using tandem mass tag (TMT) proteomics. The results showed that a total of 765 differentially expressed proteins were identified. Seventy differentially expressed proteins related to characteristic flavor were screened through Gene Ontology (GO) and Kyoto Encyclopedia of Genes and Genomes (KEGG) enrichment analysis. After integrating metabolomics data, fifteen key proteases of characteristic flavor components in BSNK-5-fermented soymilk were further identified, and free ammonia was added. In addition, there were five main formation mechanisms, including the decomposition of urea to produce ammonia; the degradation of glutamate by glutamate dehydrogenase to produce ammonia; the degradation of threonine and non-enzymatic changes to form the derivative 2,5-dimethylpyrazine; the degradation of valine, leucine, and isoleucine to synthesize isovalerate and 2-methylbutyrate; and the metabolism of pyruvate and lactate to synthesize acetate. These results provide a theoretical foundation for the improvement of undesirable flavor in *B. subtilis* BSNK-5-fermented soy foods.

## 1. Introduction

Soybeans have become the preferred raw material for the development of health-beneficial foods, due to their high-quality protein, polysaccharides, and oils, as well as a variety of bioactive substances. Microbial fermentation, as a major technological tool in the modern food industry, can not only bring rich taste, but also give food better health benefits. *Bacillus subtilis*-fermented soy foods (BFSFs) are the main fermented soybean products, including tempeh, miso, cheonggukjang, and natto. Biomolecules such as proteins, fats, and carbohydrates in soybeans are hydrolyzed to amino acids, fatty acids, and oligosaccharides via *B. subtilis*’s complex system of biological enzymes. Additionally, nattokinase, which has several thrombolytic effects, is produced [1,2]. Along with providing a number of health benefits, such as boosting immunity, regulating the intestinal flora, reducing cardiovascular disease, and improving osteoporosis, *B. subtilis* fermentation significantly increases the bioavailability of soybeans [3,4,5,6]. Our laboratory used an excellent strain, named *B. subtilis* BSNK-5, to ferment soymilk for improving the nutrient density of BFSFs and synthesizing nattokinase [7]. The nattokinase was precisely enriched, and its activity and stability were enhanced. However, with the synthesis of nattokinase, the flavor gradually deteriorated with the extension of the fermentation time [8].

The study of BFSFs’ characteristic flavor focuses on free ammonia, pyrazines [9], and branched short-chain fatty acids (BCFAs) [10,11], specifically 2,5-dimethylpyrazine (2,5-DMP), 2-methylbutyric acid, isovaleric acid, and isobutyric acid [12]. Protein degradation is an important source of volatile compounds in BFSFs, associated with BSNK-5′s complex protease system. Proteins are degraded into peptides and amino acids by the action of proteases. The amino acids are further degraded to produce volatile compounds [13]. For instance, glutaminase, glutamate dehydrogenase, asparaginase, and urease are involved in the metabolism of glutamate, aspartate, and arginine, resulting in the production of free ammonia [14]. However, the above formation pathway for volatile compounds is mainly based on simple biochemical media. Proteases associated with undesirable flavor synthesis are not systematically confirmed in BFSFs.

Proteomics is used to reveal the nutritional composition of foods, to label food allergens, and to identify the critical pathways. Tandem mass tag (TMT) proteomics has been widely used in food-related proteomics studies, offering many advantages, such as high throughput, high resolution, good reproducibility, and accurate quantification [15]. Zhihao Yang et al. used TMT-labeled quantitative proteomics to analyze the amino acid anabolic pathway of lamb’s flavor formation and found that amine oxidase, arginine succinate synthase, acetyl coenzyme A synthase, and aldolase were key enzymes involved in this process [16]. Changyu Zhou et al. analyzed the degradation and flavor enhancement mechanisms of bacon proteins inoculated with *Staphylococcus aureus* using TMT-tagged quantitative proteomics, and they found that glutamic acid, alanine, and lysine were the main factors that improved the undesirable flavor [17]. Therefore, we propose the hypothesis that protein is associated with the characteristic flavor of fermented soymilk. Due to the complexity of food fermentation, there have been fewer TMT proteomics studies on BFSFs, but the application of proteomics technology has gradually established species-specific peptide and protein databases; thus, we believe that the use of proteomics can clarify the formation mechanisms of undesirable flavors in BSNK-5-fermented soymilk.

Therefore, the objective of this study was to investigate the changes in protein expression during the BSNK-5 fermentation of soymilk (24, 48, and 84 h). The differentially expressed proteins were identified and subjected to Gene Ontology (GO) and the Kyoto Encyclopedia of Genes and Genomes (KEGG) for enrichment analysis. The metabolic pathways and key proteins associated with undesirable flavors were also screened. Through the above studies, the formation mechanisms of undesirable flavor substances in BFSFs were initially clarified.

## 2. Materials and Methods

### 2.1. Materials

*B. subtilis* BSNK-5, a probiotic strain rich in proteases, was separated and screened by our laboratory and stored at −80 °C. Soy Dongnong 690 was used as the fermentation substrate. The protease inhibitor phenyl methane sulfonyl fluoride (PMSF) was obtained from Amresco (Shanghai, China). Tris-HCl buffers (pH 6.8 and pH 8.8) were purchased from Shanghai Sangong Biotechnology Co. (Shanghai, China). Protein concentrations were determined using a BCA Assay Kit from Thermo Scientific (Shanghai, China). Sodium dodecyl sulfate (SDS) lysis buffer was purchased from Biyotime Biotechnology (Shanghai, China). Trypsin-TPCK, a type of protease, was obtained from Hualishi Reagent Co., Ltd. (Beijing, China). Triethylammonium bromide (TEAB) was supplied by Sigma-Aldrich (Shanghai, China). TMT labeling kits were purchased from Thermo Fisher Scientific (Beijing, China).

### 2.2. Preparation of Fermented Soymilk

The BSNK-5 was activated by transferring a sample to solid and liquid Luria–Bertani media according to the method of Gao et al., (2020) [8]. Briefly, the cleaned soybeans were soaked for 10 h at a ratio of 1:4 (*w*/*v*) and were ground in a blender at a ratio of 1:8 (*w*/*v*) to prepare the soymilk. The BSNK-5 was added to the soymilk bases at a dosage of 1.0% (*v*/*v*), and the fermentation continued at 37 °C and 200 rpm/min. The samples were collected at 24, 48, and 84 h. The uninoculated and unfermented soymilk (0 h) was the control sample.

### 2.3. Protein Extraction

The frozen samples were fully ground by adding liquid nitrogen. The phenol extraction solution (800 μL) and protein inhibitor PMSF were added, and the final concentration was 1 mmol/L. An equal volume of phenol-Tris-HCl solution was added to the above mixture and was continually stirred for 40 min at 4 °C. The mixture was centrifuged at 7100× *g* for 10 min, and the supernatant was collected. Five times the volume of the 0.1 mol/L ammonium acetate–methanol solution was added to the supernatant and was centrifuged to collect the protein precipitate, and the same volume of methanol as that of acetate–methanol was added to precipitate the protein again. The protein sample was dissolved in the sample lysate and centrifuged at 12,000× *g* and 4 °C for 10 min. The supernatant was collected and stored at −80 °C for further analysis. The protein concentration was determined using a BCA Assay Kit [18,19,20].

### 2.4. Protein Molecular Weight Analysis using Sodium Dodecyl Sulfate–Polyacrylamide Gel Electrophoresis (SDS-PAGE)

The extracted protein (5 mg/mL) was dispersed in deionized water. The protein solution (30 μL) was mixed with 20 μL of 150 mM Tris-HCl buffer solution and was heated at 100 °C for 30 s. Then, 10 μL of the mixture was loaded onto the electrophoresis gel (5% stacking and 12% separating gel) (Beijing Solei Bao Technology Co., Ltd. Beijing, China), which was run at 80 V for the stacking gel and 120 V for the separating gel using an electrophoresis cell (Mini II, Bio-Rad Laboratories, Hercules, CA, USA). After electrophoresis, the gel was stained with Coomassie Brilliant Blue for 30 min. The gels were diffusion-destained by washing them 5 times in 95% ethanol and acetic acid (3:2, *v*/*v*) until the background of the gel became clear. The diffusion-destained gel was scanned using a gel imaging system (GEL-Doc 2000; Bio-Rad Laboratories, Hercules, CA, USA).

### 2.5. Proteolytic Enzymolysis

In this stage, 100 μg of protein was diluted to an appropriate concentration, and dithiothreitol (DTT) was added to the diluted protein solution to achieve a final concentration of 5 mmol/L. The mixture was then incubated at 55 °C for 30 min and was subsequently cooled to room temperature. Equal volumes of iodoacetamide were added to bring the final concentration to 10 mmol/L, which required incubation in the dark for 15 min. Subsequently, acetone was added to the above samples, which were then stored at −20 °C overnight. The protein precipitate was collected after centrifugation and was left at room temperature for 2–3 min to allow the acetone to evaporate. Finally, 100 μL of 200 mmol/L triethylammonium bicarbonate (TEAB) buffer and 2 μL of Trypsin-TPCK (1 mg/mL) were added, with the reaction proceeding overnight at 37 °C. The resultant enzyme-digested samples were then lyophilized and stored at −80 °C for preservation purposes [21,22].

### 2.6. Protein Labeling

Peptide labeling was performed according to existing methods [23,24]. The samples were re-dissolved in 100 mmol/L TEAB buffer solution and mixed well. Then, 41 μL of equilibrated TMT reagent was added to 30 μL of the above solutions. The mixture was incubated at room temperature for 1 h and the reaction was terminated by the addition of hydroxylamine for 15 min. The labeled samples were then lyophilized and stored at −80 °C.

### 2.7. Protein Fractionation

Labeled peptides were separated using high-pH reversed-phase high-performance liquid chromatography (RP-HPLC, Agilent, Santa Clara, CA, USA) with an Agilent Zorbax Extend-C18 narrow-bore column (dimensions: 2.1 mm × 150 mm; particle size: 5 μm) [25,26]. The separation was obtained with a gradient of two mobile phases—A (water containing 2% acetonitrile) and B (acetonitrile containing 90% water)—at a 300 μL/min flow rate. The gradient was started at 2% B and was ramped to 5% in 8 min, from 6 to 25% B in 40 min, from 26 to 40% B in 12 min, holding at 90% B for 10 min, and going down to 2% B in 1 min, before equilibration for 5 min for the next injection. The absorbance was measured at 210 nm and 280 nm. Samples were collected from 8 to 60 min, for a total of 15 peptide groups. The separated samples were lyophilized and stored at −80 °C.

### 2.8. Peptide Profiling Analysis

For peptide profiling, liquid chromatography–tandem mass spectrometry (LC-MS/MS) analysis was performed using a Triple TOF 5600 System (AB SCIEX, Coppell, TX, USA). The peptides were first loaded onto a C18 trap column (100 μm × 200 mm, RP-C18 column, Thermo Fisher Scientific, Waltham, MA, USA) and were then eluted into a C18 analytical column (75 μm × 150 mm, RP-C18 column, Thermo Fisher Scientific, USA). Mobile phase A (0.1% formic acid) and mobile phase B (80% ACN and 0.1% formic acid) were used to establish a 60 min separation gradient. A constant flow rate was set at 300 nL/min. The resulting MS files were analyzed using the Proteome Discoverer 2.4 software (Thermo Fisher). The peptide mass tolerance was set to 10 ppm; the MS/MS tolerance was set to 0.02 Da.

### 2.9. Bioinformatics Analysis

Data were analyzed using Proteome Discover 2.4 (Thermo Fisher, Waltham, MA, USA) software. The credible proteins were screened from original data under Score Sequest HT > 0 and unique peptide ≥ 1. Subsequently, the differentially expressed proteins were subjected to sequence comparison, GO analysis, and KEGG analysis for functional annotation, and the relevant databases included UniProt, KEGG, and GO. The significance was analyzed by means of the hypergeometric distribution test and is expressed as a p-value. Cluster analysis heatmaps and Venn analysis plots were also made for the comparison of differences in the group data. In addition, key proteins associated with undesirable flavor substances were selected for subsequent focused research and validation directions.

## 3. Results

### 3.1. Protein Concentration and Molecular Weight Distribution

**Figure 1:**
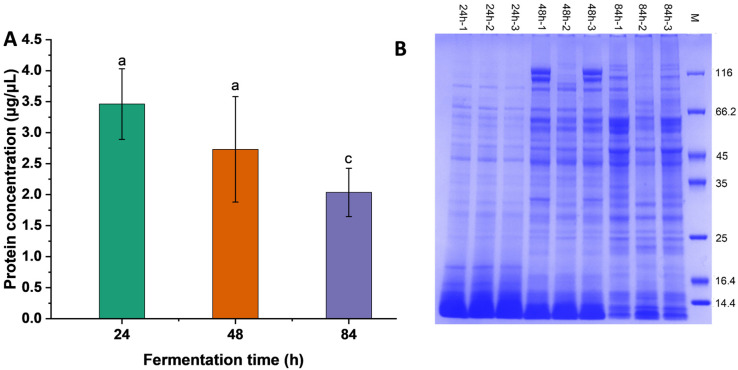
The concentration and SDS-PAGE of sample proteins. (**A**) The protein concentration; (**B**) SDS-PAGE of proteins with different fermentation times. The different letters represented the significant difference (*p* < 0.05).

As shown in Figure 1A, with the extension of the fermentation time, the protein concentration of BSNK-5-fermented soymilk was decreased. The molecular weight distribution of BSNK-5-fermented soymilk was measured using SDS-PAGE (Figure 1B). The protein bands of each sample were clear and evenly distributed, with no degradation, and were relatively parallel to one another at the same time. The results indicated that the protein extraction of fermented soymilk samples at different times was good and could be used for subsequent analysis. The lighter band appeared at 24 h, indicating that the heat treatment prompted the aggregation behavior to form high-molecular-weight protein molecules, and the protein was degraded further with increasing fermentation time, showing deeper bonds.

### 3.2. Identification of Proteins using Quantitative Proteomics Analysis

**Figure 2:**
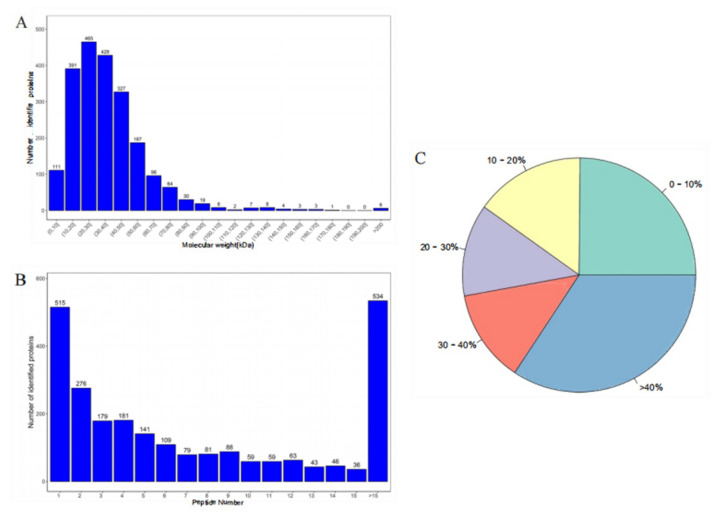
(**A**) The distribution of protein molecular weight; (**B**) the distribution of peptide numbers; and (**C**) the extent of peptide sequence coverage.

The mass spectrometry database was utilized for protein identification in the BSNK-5-fermented soymilk samples (Figure 2). The results showed that the majority of the proteins fell within the 10–50 kDa range. Approximately 78.55% of the proteins exhibited a polypeptide count between 0 and 10 kDa, indicating that the samples were well digested. Furthermore, the higher the peptide number, the more reliable the protein. The extent of peptide sequence coverage can also reflect the overall accuracy of the identification results [27]. The proteins that were identified exhibited low sequence coverage, as more than 75% of them displayed more than 10% sequence coverage, and 59.92% of them displayed more than 20% sequence coverage. These findings suggest that the data utilized in this investigation exhibit a high degree of reliability and possess a significant level of statistical certainty.

### 3.3. Protein Identification

**Figure 3:**
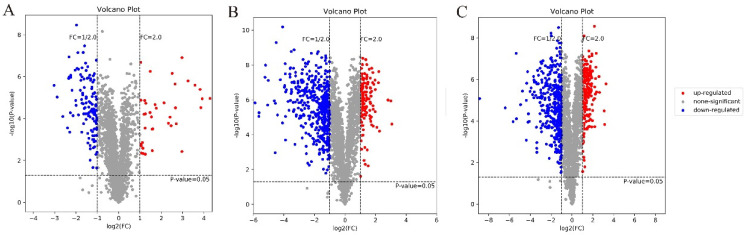
The volcano plots of differentially expressed proteins in different groups. (**A**) Comparison groups of 24 h and 48 h fermented soymilk (group 1); (**B**) 24 h and 84 h fermented soymilk (group 2); (**C**) 48 h and 84 h fermented soymilk (group 3).

Quantitative analysis was performed via two-by-two comparisons to obtain the following comparison groups: 24 h and 48 h fermented soymilk (group 1), 24 h and 84 h fermented soymilk (group 2), and 48 h and 84 h fermented soymilk (group 3). The significance of differences between samples was initially assessed using *t*-tests, generating *p*-values. The samples were screened according to the peak intensity of the label ion, and the fold-change (FC) was used to evaluated the expression level of a protein between samples. The volcano plots visually depict the differential protein distribution in the three comparison groups. The red dots indicate significant upregulations (FC ≥ 2, *p* < 0.05), while the blue dots indicate significant downregulations (FC < 0.5, *p* < 0.05), and the gray dots indicate no significant changes in regulation (Figure 3). There were 159 differentially expressed proteins in group 1; the 38 red dots in the volcano plot indicate differentially upregulated proteins, while the 121 blue dots indicate differentially downregulated proteins (Figure 3A). There were 568 differentially expressed proteins in group 2; the 138 red dots in the volcano plot indicate differentially upregulated proteins, while the 430 blue dots indicate differentially downregulated proteins (Figure 3B). There were 586 differentially expressed proteins in group 3; the 217 red dots in the volcano plot indicate differentially upregulated proteins, while the 369 blue dots indicate differentially downregulated proteins (Figure 3C). Notably, group 3 exhibited the highest number of differentially expressed proteins, suggesting that the number of such proteins continued to increase with fermentation time, gradually stabilizing.

**Figure 4:**
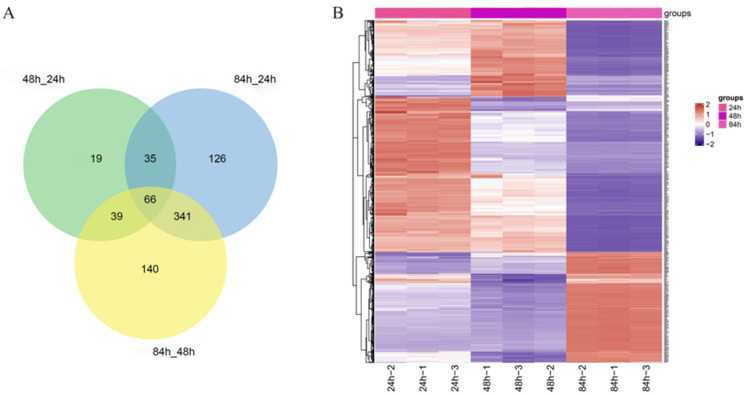
(**A**) Venn diagram and (**B**) hierarchical clustering analysis of differentially expressed proteins.

For the screening data for the three comparison groups, the differential protein characteristics and commonalities in each group were analyzed with Venn diagrams, with different colors representing different groups, and the numbers in Figure 4A denote the numbers of intersecting proteins, as well as the numbers of proteins specific to each group. A total of 765 differentially expressed proteins were identified from the BSNK-5-fermented soymilk. Furthermore, these differentially expressed proteins were subjected to unsupervised hierarchical clustering in order to construct a heatmap based on the differential expression patterns with log 2 values (infected/control) and fold-changes. An unsupervised hierarchical clustering analysis of these 765 differentially expressed proteins was performed based on the R language for visualizing the multiplicity and distribution of differences (Figure 4B). Each protein was clustered in the horizontal direction, and each sample was clustered in the vertical direction. The different colors represent the amount of protein expression, and a change from red to blue indicates a decrease in expression. Differentially expressed proteins could be further classified into three categories using the hierarchical clustering analysis of fermented soymilk at 24, 48, and 84 h. The significant differences among the three comparison groups suggest that the fermentation time influenced the protein expression in BSNK-5-fermented soymilk.

### 3.4. Global Analysis of Differentially Expressed Proteins

#### 3.4.1. GO Enrichment Analysis of Differentially Expressed Proteins

**Figure 5:**
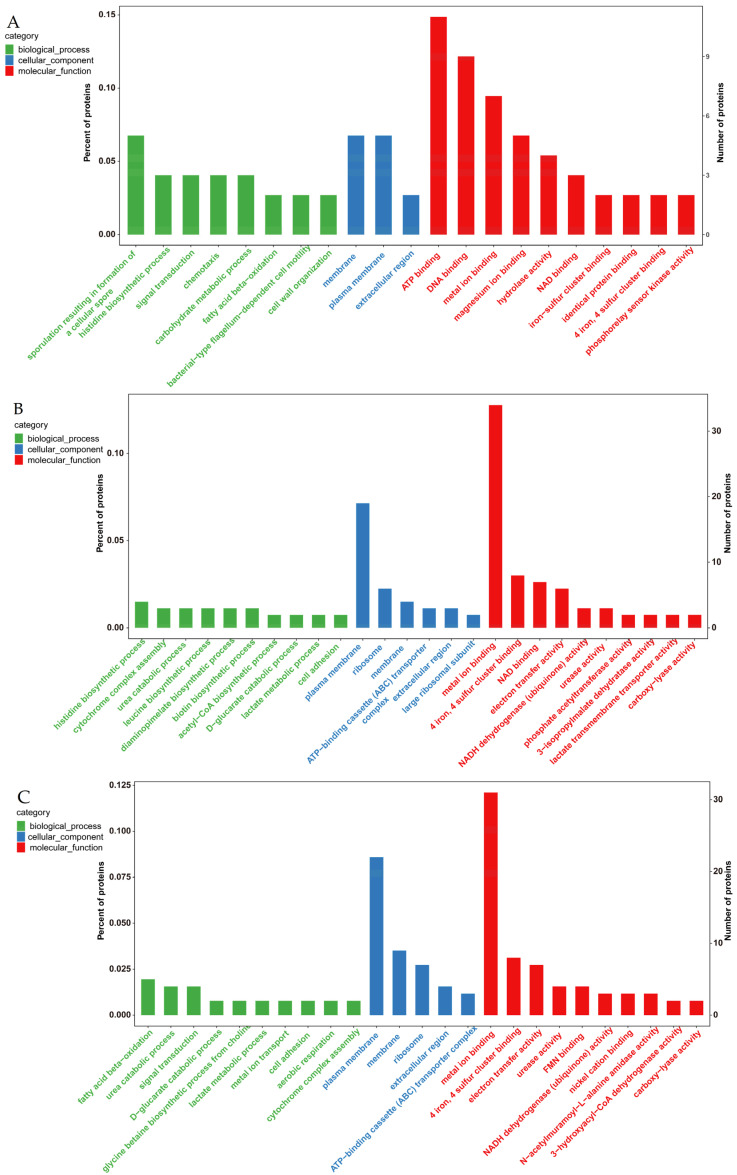
GO enrichment analysis for different groups: (**A**) 1–2 comparison group: 24 h vs. 48 h; (**B**) 1–3 comparison group: 24 h vs. 84 h; (**C**) 2–3 comparison group: 48 h vs. 84 h.

The differentially expressed proteins were annotated and enriched using GO to explain the biological functions that the proteins perform in organisms, tissues, or cells. All enriched GO terms were classified into three ontological categories of biological processes (BPs), molecular functions (MFs), and cellular components (CCs) (Figure 5). In group 1, the differentially expressed proteins underwent binding of the same proteins, cations, and metal ions as in the MF category. In the BP category, the differentially expressed proteins played a role in the regulation of fatty acids and carbohydrates’ oxidative metabolism, as well as signaling. In the CC category, “cellular parts”, “cell membrane”, “cytoplasm”, and “extracellular regions” stood in for the differentially expressed proteins. In group 2, a greater number of differentially expressed proteins—primarily ion-bound, but also connected to enzyme function—including urease, NADH dehydrogenase, and hydroxyacetate were identified in the MF category. Differentially expressed proteins in the BP category were mostly grouped for lactate metabolism; urea and glucose catabolism; and histidine, leucine, and acetyl coenzyme A production. In the CC category, there were more ribosomes. In group 3, the distribution of differentially expressed proteins in the classifications was similar to that found in group 2, but the proteins changed differently in the MF category, mainly with the synthesis of betaine and the emergence of systematic processes of aerobic respiration, cell adhesion, and metal ion migration. These results demonstrated that the functional distribution of proteins tended to be the same in late soymilk fermentation but had a greater impact on the metabolism of substances in biological processes.

#### 3.4.2. KEGG Analysis of Differential Proteins

**Figure 6:**
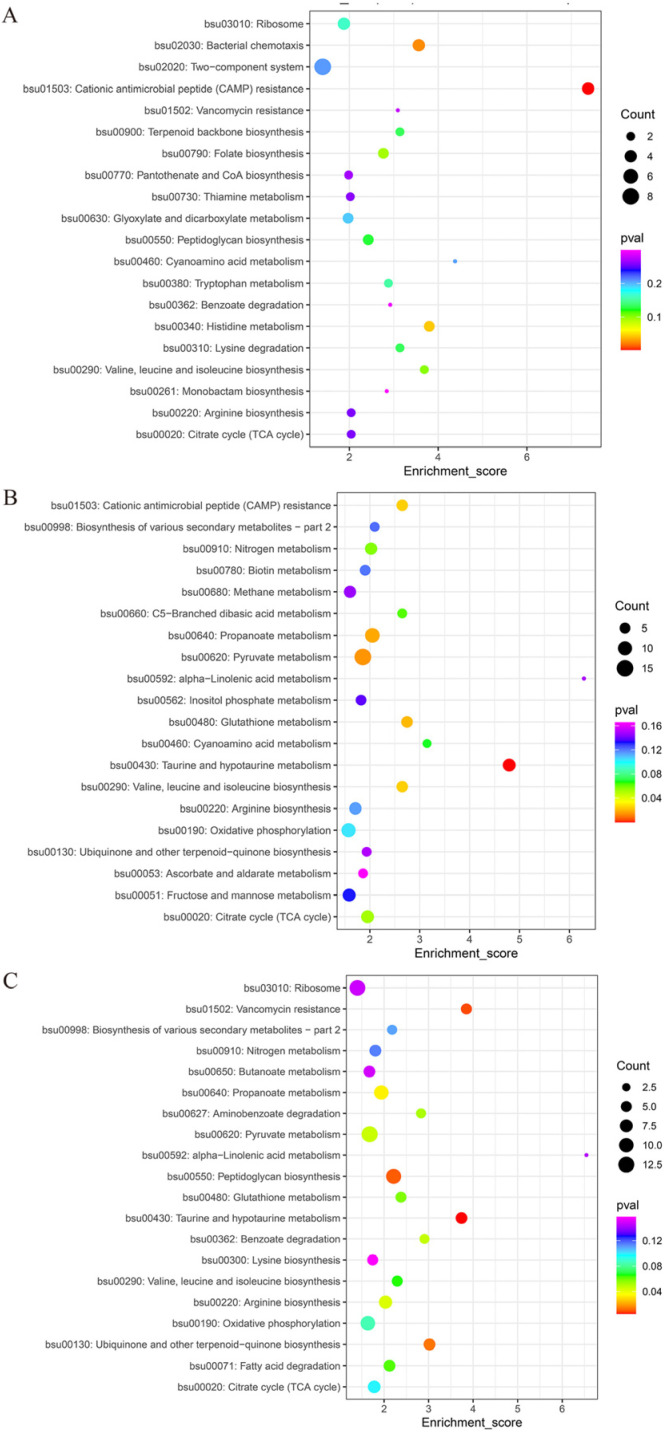
The KEGG bubble charts for different groups: (**A**) 1–2 comparison group: 24 h vs. 48 h; (**B**) 1–3 comparison group: 24 h vs. 84 h; (**C**) 2–3 comparison group: 48 h vs. 84 h.

KEGG is a public database that systematically analyzes the metabolic pathways of proteins in organisms, and proteins identified from metabolic pathway information can be annotated and enriched using the KEGG database. As illustrated in Figure 6, differentially expressed proteins in all three comparison groups were extensively involved in many biological processes, with a total of 43 KEGG-enriched pathways. Differentially expressed proteins were specifically involved in the TCA cycle, nitrogen metabolism, methane metabolism, fructose and mannose metabolism, pyruvate metabolism, folate biosynthesis, ascorbate and aldarate metabolism, benzoate degradation, inositol phosphate metabolism, lysine degradation, and ubiquinone and other terpenoid–quinone biosynthesis. The metabolic pathways of groups 2 and 3 were similar, including anabolism of functional components such as taurine, glutathione, and α-linolenic acid. Moreover, the metabolic pathway of group 1 was significantly different from those of the other groups. These results showed that the participation of differentially expressed proteins in biological processes was affected by fermentation time, where the distribution of differentially expressed proteins in the metabolic pathways gradually converged with the extension of the fermentation time.

### 3.5. Selection of Differentially Expressed Proteins Associated with Characteristic Flavor Formation

Since the volatile components associated with characteristic flavor in natto products are mainly free ammonia, pyrazines, and BCFAs [10], the screening of differentially expressed proteins associated with characteristic flavor was subjected to the synthesis of the above compounds (Table 1). A total of 10 metabolic pathways were screened, and 69 differentially expressed proteases associated with undesirable flavor synthesis were initially identified according to the annotation results for differentially expressed proteins and KEGG enrichment pathways. There were eight metabolic pathways in the metabolism of alanine, aspartate, and glutamate; five in the degradation metabolism of leucine, isoleucine, and valine; six in the metabolism of leucine, isoleucine, and valine synthesis; nine in glycine, serine, and threonine metabolism; four in arginine and proline metabolism; seven in arginine anabolism; seven in purine metabolism; four in the pentose phosphate pathway; seven in the TCA cycle; and twelve in pyruvate metabolism. Furthermore, each protein was involved in differential metabolism crossover. Differentially expressed proteins associated with characteristic flavor were mainly found in groups 2 and 3, such as glutamate dehydrogenase, urease, ornithine transaminase, lipoyl dehydrogenase, and other proteases, whereas they were extremely rare in group 1, suggesting that metabolism associated with characteristic flavor was more vigorous in the later stages of fermentation.

## 4. Discussion

### 4.1. Metabolic Pathways of Amino Acids

The synthesis of characteristic flavor substances in BSNK-5-fermented soymilk was mainly related to amino acid metabolism. In *B. subtilis*, at least five metabolic pathways have been implicated in ammonia production. Glutamate produces α-ketoglutarate and free ammonia through glutamate dehydrogenase (GDH) [28]. Glutamine was catalyzed by glutaminase to produce glutamate and free ammonia [29]. Aspartic acid was degraded to fenugreek and free ammonia under the action of asparaginase [30]. Asparagine synthesized aspartic acid and free ammonia under the action of asparaginase -. Arginine is metabolized through catabolism and enters the urea cycle, where urease catalyzes the production of free ammonia from urea [31]. Ammonia-producing differentially expressed proteins associated with aspartic acid or asparagine were not screened in the TMT-based proteomic analysis. Nineteen differentially expressed proteins were involved in arginine metabolism (including proline and purine), of which four were associated with ammonia production via urea degradation. Additionally, the content of urease was increased in the 48 h fermented soymilk, indicating that ammonia-producing metabolism in urea was one of the major contributors to the production of undesirable flavors in BSNK-5-fermented soymilk. There are two types of glutamate anabolism in microorganisms, but GDH in *B. subtilis* was only involved in catabolism, not anabolism; therefore, glutamate synthesis was achieved through the glutamine oxoglutarate aminotransferase (GOGAT) and glutamine synthetase (GS) pathways [32]. The rocG gene encoding GDH exhibits high activity due to the high protein and nitrogen content of soybeans [33], and the overexpression of the rocG gene leads to high levels of expression of the gltAB gene encoding GOGAT [34]. Furthermore, the synthesis of GDH was inhibited by glucose [35]. Interestingly, the NAD^+^ (NADP^+^) produced during lysine synthesis acts as a coenzyme and catalyzes the formation of free ammonia. In BSNK-5 fermentation, the levels of the GDH protein initially increased at 48 h and then decreased significantly, which was attributed to the overexpression of rocG in the pre-fermentation phase, as well as glucose inhibiting the synthesis of GDH in the late fermentation phase. The results suggested that GDH played a role in glutamate deamination, contributing to the accumulation of ammonia in soymilk. It can be inferred that arginine and glutamate metabolism have a more pronounced impact on the characteristic flavor during the early and middle stages of fermentation, with less influence in the later stages.

### 4.2. Metabolic Pathways of Pyrazine Compounds

The pyrazines detected in *B. subtilis* fermentation were tetramethylpyrazine (TTMP) and 2,5-DMP, where the TTMP molecule was generated by the combination of ethyl-coupling microbial sugar metabolites with free ammonia from amino acid metabolism. The precursor substance of 2,5-DMP is threonine, which involves L-threonine 3-dehydrogenase (TDH) and three non-enzymatic reactions, and the synthesis mechanism of 2,3,5-trimethylpyrazine (2,3,5-TMP) is similar to that of 2,5-DMP [36,37]. The effective use of oxygen caused the overproduction of pyrazines, with adverse effects on product flavor above their thresholds. However, the mechanism of alkylpyrazine synthesis by microorganisms is still poorly understood, and no clear metabolic mechanism has been identified.

The 2,5-DMP precursor, threonine, is produced by the metabolism of aspartic acid, which involves the production of homoserine catalyzed by aspartate kinase (AK) and aspartate semialdehyde dehydrogenase (ACADH), followed by the synthesis of threonine by homoserine kinase (HK) and threonine synthase (TS). TDH catalyzes the formation of aminopropanone from threonine or its degradation to 2-ketobutyrate, but due to structural instability, it can spontaneously decarboxylate to form aminopropanone, which can then be synthesized via non-enzymatic reactions such as the oxidation or cycloaddition of the aminopropanone to 2,5-DMP. The increase in 2,5-DMP content may be due to the indirect production of aminopyruvic acid from pyruvic acid via the expression of enzymes in serine and glycine metabolism. TTMP was not detected, indicating that synthesis of the ethyl-coupling precursor was blocked. Ethyl coupling is obtained from pyruvate catalyzed by ALS and acetolactate decarboxylase (ALDC). ALDC, a protease, was not screened in the TMT protein assay results, and the relative expression of ALS was always decreasing, while the relative expression of β-dehydrogenase (ACDA) increased and then decreased. Its degradation activity was higher than its synthesis activity, suggesting that the degradation system of ethylenic amphiphiles was active in the early stage, and the process was weakened in the later stage due to the decrease in ethylenic amphiphiles.

### 4.3. Metabolic Pathways of BCFAs and Acetic Acid

BCFAs such as isovaleric acid, isobutyric acid, and 2-methylbutyric acid contribute to natto’s characteristic flavor [38,39]. The BCFAs produced are mainly associated with the metabolism of branched-chain amino acids—namely, isoleucine, leucine, and valine—during microbial fermentation [40]. Branched-chain amino acids first catalyze the irreversible oxidative decarboxylation of branched-chain α-keto acids (including α-ketoisocaproic acid, α-keto-β-methylvaleric acid, and α-ketoisopentanoic acid) through the activities of branched-chain aminotransferase and branched-chain α-keto acid dehydrogenase complexes, similar to the activities of the branched-chain alpha-keto dehydrogenase complex dihydrolipoamidoacetyltransferase (BCKDC-DLAT) complex proteases identified in the present study, to form 2-methylbutyric, isobutyric, and isovaleric acids, respectively, of the coenzyme A derivatives, which are then stretched by the well-characterized free fatty acid biosynthesis (FAS II) system to form BCFAs [41,42]. Ketoacid reduction isomerase (KARI), dihydroxy acid dehydratase (DHAD), and BCKDC-DLAT proteases were associated with branched-chain amino acid synthesis and degradation, in which BCKDC was a key rate-limiting enzyme in the branched-chain amino acid catabolic pathway [43]. The expression of BCKDC-DLAT showed a significant increase, and the change was more obvious at the late fermentation stage, indicating that the main source of undesirable flavor substances in soymilk was BCFAs, dominated by 2-methylbutyric acid. In the TMT proteomics analysis, it was shown that the expression level of acetokinase (ACK) was decreased. Meanwhile, the synthesis of the ethyl coupling was blocked from competing for lactic acid, causing excess acetic acid in BSNK-5-fermented soymilk, resulting in an irritating sour taste. The results demonstrated that acetic acid is also one of the substances that causes the characteristic flavors in BSNK-5-fermented soymilk.

## 5. Conclusions

The protein expression profile of BSNK-5 during soymilk fermentation was identified using TMT-labeled quantitative proteomics technology, and a total of 765 differentially expressed proteins were identified. Through GO and KEGG enrichment analysis, the differentially expressed proteins were found to be mainly involved in molecular functions and carbon and amino acid metabolism pathways. Seventy differentially expressed proteins associated with undesirable flavors were screened, and fifteen key proteases associated with the synthesis of undesirable flavors were identified. Seven metabolic pathways associated with the synthesis of undesirable flavor substances were identified, including the pentose phosphate pathway; the TCA cycle; the urea cycle; glutamate degradation metabolism; threonine degradation metabolism; pyruvate metabolism; and leucine, isoleucine, and valine degradation metabolism.

## Figures and Tables

**Table 1:** Differentially expressed proteins related to the synthesis of undesirable flavor substances.

Key Process	Accession	Gene Name	Protein Description	Group 1 FC	Group 2 FC	Group 3 FC
Alanine, aspartate, and glutamate metabolism	A0A6H0H898	*pruA*	Adenylosuccinate synthetase		0.17	0.12
	G4EZA6	*purF*	Amidophosphoribosyl transferase		0.38	0.33
	A0A6H2KH29	*ald*	Alanine dehydrogenase		0.34	0.23
	P94427	*gabT*	Probable 4-aminobutyrate aminotransferase		0.14	0.12
	A0A199WDG9	*rocG*	Glutamate dehydrogenase		0.19	0.17
	A0A6H2K2D7	*gltA*	Glutamate synthase (NADPH) large chain		0.37	0.40
	A0A6H2K2B8	*gltB*	NADPH small chain		0.49	
	A0A1B2AVT2	*carB*	Carbamoyl-phosphate synthase large chain		0.20	0.21
Valine, leucine, and isoleucine degradation	A0A6I4DBI8	*bfmBB*	Branched-chain alpha-keto dehydrogenase complex dihydrolipoyllysine-residue (2-methylpropanoyl) transferase		2.29	2.02
	A0A6H2KKS5	*iolA*	Methylmalonate/inositol-semialdehyde dehydrogenase		0.44	0.49
	G4EYT5	*BSSC8_35150*	Dihydrolipoyl dehydrogenase		0.13	0.17
	A0A6H0H9I7	*HCN55_12915*	BSU23920		0.23	0.19
	A0A6H0H6V2	*HCN55_17715*	Acetyl-CoA C-acyltransferase		0.39	0.23
Valine, leucine, and isoleucine biosynthesis	P37253	*ilvC*	Ketol-acidreductoisomerase (NADP(+))	0.43	0.10	0.22
	A0A6I4D8C8	*ilvD*	Dihydroxy-acid dehydratase		0.41	0.45
	P05645	*leuB*	3-isopropylmalate dehydrogenase	0.35	0.15	0.42
	G4EST7	*leuC*	3-isopropylmalate dehydratase large subunit		0.21	0.35
	P94568	*leuD*	3-isopropylmalate dehydratase small subunit		0.30	
	A0A6H0H8M1	*alsS*	Acetolactate synthase AlsS		0.32	0.27
Glycine, serine, and threonine metabolism	Q04797	*asd*	Aspartate-semialdehyde dehydrogenase		0.45	
	A0A1B2B4F9	*kbl*	8-amino-7-ketopelargonate synthase		0.47	0.34
	O31776	*tdh*	L-threonine 3-dehydrogenase		0.20	0.20
	A0A6H2K6M6	*yvcT*	Glyoxylate reductase YvcT		0.44	
	P08495	*lysC*	Aspartokinase 2		0.26	0.41
	A0A199WF35	*gbsA*	Betaine aldehyde dehydrogenase			2.46
	P71017	*gbsB*	Choline dehydrogenase		2.70	2.64
	A3F3S8	*trpA*	Tryptophan synthase alpha chain			2.11
	A0A6H2K323	*trpB*	Tryptophan synthase beta chain		2.60	3.69
Arginine and proline metabolism	A0A6H0H1Z3	*proA*	Glutamate-5-semialdehyde dehydrogenase		0.37	0.29
	P21885	*speA*	Arginine decarboxylase		0.31	0.36
	G4EP73	*rocD*	Ornithine aminotransferase		0.27	0.26
	P39138	*rocF*	Arginase			0.47
Arginine biosynthesis	A0A6H2KBK9	*argC*	N-acetyl-gamma-glutamyl-phosphate reductase		0.27	0.40
	A0A6H2K084	*argF*	Ornithine carbamoyltransferase		0.12	0.09
	O34984	*yodQ*	Uncharacterized metallohydrolase YodQ	0.27	0.39	1.44
	P77837	*ureC*	Urease subunit alpha		0.14	0.17
	P71035	*ureB*	Urease subunit beta		0.18	0.18
	P75030	*ureA*	Urease subunit gamma		0.07	0.12
	G4EQB1	*BSSC8_05410*	Urea amidohydrolase	2.77		0.20
Purine metabolism	A0A6H0GZI4	*HCN55_03695*	Adenine deaminase		0.46	0.27
	G4EWI5	*sat*	Sulfate adenylyltransferase	0.49	0.18	0.38
	A0A6H2JX42	*dgk*	Deoxyguanosine kinase DGK		0.38	
	A0A1B2B5S8	*rdgB*	dITP/XTP pyrophosphatase			0.36
	A0A6H2K5V9	*pucH*	Allantoinase		0.16	0.18
	O32141	*pucL*	Uric acid degradation bifunctional protein PucL		0.31	0.45
	A0A6H2KDK2	*nrdEB*	Ribonucleotide reductase of class Ib (Aerobic)		2.04	1.79
Pentose phosphate pathway	A0A6H0GYQ1	*HCN55_02205*	SDR family oxidoreductase	0.39	0.93	2.34
	A0A6H2KGT5	*zwf*	NADP-dependent glucose-6-phosphate 1-dehydrogenase		0.34	0.45
	G4EU42	*BSSC8_18720*	6-phosphogluconate dehydrogenase, decarboxylating		0.0	0.35
	Q03224	*glpX*	Fructose-1,6-bisphosphatase class 2		2.06	1.81
TCA cycle	A0A1B2BE85	*pckA*	Phosphoenolpyruvate carboxykinase (ATP)		2.34	2.23
	A0A6H0H2Z7	*odhB*	2-oxoglutarate dehydrogenase complex dihydrolipoyllysine-residue succinyltransferase	2.37	3.70	
	A0A6H0H5Z3	*sdhA*	Succinate dehydrogenase flavoprotein subunit		0.42	0.37
	A0A6H2K2M0	*pycA*	Pyruvate carboxylase		2.20	2.47
	A0A6I4DHN9	*mmgD*	2-methylcitrate synthase	0.49		
	G4ESR9	*BSSC8_13990*	Succinate dehydrogenase (quinone)		0.43	0.43
	G4EWR2	*BSSC8_27920*	Pyruvate carboxylase		2.03	2.09
Pyruvate metabolism	A0A199WLB6	*ackA*	Acetate kinase		0.45	0.49
	A0A6H0H0J6	*acoC*	Acetoin dehydrogenase complex dihydrolipoyllysine-residue acetyltransferase		0.11	0.08
	A0A6H0H4J8	*accC*	Acetyl-CoA carboxylase biotin carboxylase subunit		2.16	2.25
	A0A6H0H6N9	*acsA*	Acetate--CoA ligase		2.24	2.59
	G4EQ01	*BSSC8_04310*	Phosphotransacetylase		0.41	0.42
	G4EQ65	*BSSC8_04950*	Malate dehydrogenase (oxaloacetate-decarboxylating)		0.35	0.31
	G4EQR2	*BSSC8_06920*	Putative phosphotransferase		2.04	3.04
	G4F094	*ldh*	L-lactate dehydrogenase		0.09	0.12
	G9LQ56	*yngH*	Acetyl-CoA carboxylase biotin carboxylase subunit	0.46		
	O35031	*acyP*	Acylphosphatase			0.43
	P39646	*pta*	Phosphate acetyltransferase		0.37	
	P54501	*yqgX*	Probable metallo-hydrolase YqgX		0.39	0.55

Note: Group 1 indicates the 24 h vs. 48 h fermented soymilk comparison group. Group 2 indicates the 24 h vs. 84 h fermented soymilk comparison group. Group 3 indicates the 48 h vs. 84 h fermented soymilk comparison group.

## Data Availability

The original contributions presented in the study are included in the article, further inquiries can be directed to the corresponding author.
